# Microstructured soft devices for the growth and analysis of populations of homogenous multicellular tumor spheroids

**DOI:** 10.1007/s00018-023-04748-1

**Published:** 2023-03-16

**Authors:** Ottavia Tartagni, Alexandra Borók, Emanuela Mensà, Attila Bonyár, Barbara Monti, Johan Hofkens, Anna Maria Porcelli, Giampaolo Zuccheri

**Affiliations:** 1 Department of Pharmacy and Biotechnology, University of Bologna, Via San Donato, 19/2, 40127 Bologna, Italy; 2 Department of Electronics Technology, Budapest University of Technology and Economics, Budapest, Hungary; 3 Interdepartmental Center for Industrial Research on Health Sciences and Technologies, University of Bologna, Bologna, Italy; 4 Department of Chemistry, KU Leuven, 3001 Leuven, Belgium; 5 S3 Center, Institute of Nanoscience, Italian National Research Council, Modena, Italy

**Keywords:** Microwells, Microtissues, Spheroid, Cancer models, 3D cell culture, Drug screening, High content screening, High-throughput screening

## Abstract

Multicellular tumor spheroids are rapidly emerging as an improved in vitro model with respect to more traditional 2D culturing. Microwell culturing is a simple and accessible method for generating a large number of uniformly sized spheroids, but commercially available systems often do not enable researchers to perform complete culturing and analysis pipelines and the mechanical properties of their culture environment are not commonly matching those of the target tissue. We herein report a simple method to obtain custom-designed self-built microwell arrays made of polydimethylsiloxane or agarose for uniform 3D cell structure generation. Such materials can provide an environment of tunable mechanical flexibility. We developed protocols to culture a variety of cancer and non-cancer cell lines in such devices and to perform molecular and imaging characterizations of the spheroid growth, viability, and response to pharmacological treatments. Hundreds of tumor spheroids grow (in scaffolded or scaffold-free conditions) at homogeneous rates and can be harvested at will. Microscopy imaging can be performed in situ during or at the end of the culture. Fluorescence (confocal) microscopy can be performed after in situ staining while retaining the geographic arrangement of spheroids in the plate wells. This platform can enable statistically robust investigations on cancer biology and screening of drug treatments.

**Supplementary Information:**

The online version contains supplementary material available at 10.1007/s00018-023-04748-1.

## Introduction

It was estimated that cancer killed almost 10 million people globally in 2020, making it the second leading cause of death [1]. Efforts to produce adequate cancer treatments are ongoing; nevertheless, clinical translation of anti-cancer drugs must overcome various hurdles from the early discovery phases to a successful translation [2, 3]. Currently, preclinical studies follow a standard pipeline based on drug efficacy tests for in vitro studies on 2D cell monolayers, murine in vivo models, and toxicity studies on two animal species for regulatory purposes and clinical trials. Generally, a two-dimensional (2D) cell culture system differs substantially from the environment that cells experience in vivo; therefore, the validity of acquiring data from 2D systems for drug screening is limited and can be misleading [4, 5]. A greater understanding of the relevance of cellular interactions in the setting of their unique microenvironment is likely to be responsible for the current increase in interest and exploration of 3D cell culture, particularly in cancer research. Multi-cellular tumor spheroids (MTS) better simulate the complex in vivo milieu and recapitulate cellular behaviors similar to the physiological condition. Multicellular tumor spheroids are nowadays the most extensively used 3D tumor model in preclinical studies, and various techniques have been developed for their production [6–8]. The hanging-drop method or the use of ultra-low attachment multi-well plates is currently widespread and accessible MTS production methods. However, since just a single spheroid can be generated in a plate well or in a single droplet, the number of MTS that can be produced using these two techniques is quite limited [9–12]. Alternatively, large amounts of MTS may be produced by different techniques including 3D bioprinting or dispersion seeding in droplets of the matrix: the resulting homogeneity of MTS is poor [13], as is the control and homogeneity of their shape. Even though these methods have been used in screening platforms for pharmacological treatments [13–17], their major drawback is the heterogeneity of MTS, which makes these techniques unsuitable for standardized, reproducible high-throughput drug testing. The heterogeneity or the small number of employed spheroids can significantly impact the result of assays of the pharmacological effectiveness and toxicity. For instance, larger spheroids can contain more quiescent or hypoxic cells in their cores and those could be shielded from drug penetration or simply more resistant, while smaller MTS could be more exposed to the environment and thus more sensitive to treatments. Standardized spheroid fabrication processes minimize data variability and improve the clinical value of experimental data produced from 3D culture systems [18]. Microstructured multi-well culturing devices have been made commercially available in recent years. In these, numerous sub-millimeter microwells act as separate cradles for splitting the cell seeding evenly and providing physical support for the development of homogeneously sized and shaped MTS. This made it possible to generate large numbers of 3D cellular spheroids that possess the in vivo characteristics of cancers, pancreatic islets, liver, and embryoid bodies [19–21]. The power of this class of techniques lies in the simple mass production of extremely homogeneous spheroids. However, these microstructured platforms still require improvements concerning specimens handling, high-resolution imaging, tracking of individual spheroids, and the possibility to perform complex characterization procedures easily to fully harness the power of this type of platform. Often, these culturing devices are only used for seeding or growing the MTS, while all subsequent characterization procedures are performed ex situ, making procedures more complicated and time consuming. Additionally, it is nowadays ever more recognized that the mechanical environment where cells grow is key to defining their characteristics and behavior [22–24] and so there is a need for effective 3D cell culture devices that could modulate the mechanical properties of the culture environment avoiding hard plastics, while still taking advantage of methods that produce large amounts of homogeneous MTS. In this paper, the simple fabrication and the use of a microstructured 3D cell culture platform are reported. It was tailored to generate large populations of homogeneous spheroids from different cell lines. The proposed devices may be used to quantify growth or size reduction after drug treatments also using standard viability assays. Spheroids could be generated using methods that use or do not use scaffolds. Complex protocols such as live/dead staining and immunofluorescence could be performed in a loss-less fashion in the presented devices without the need for spheroid harvesting and handling. As a result, this approach preserves the integrity of the spheroids without any damage due to harvesting. Since microwells are made of polydimethylsiloxane (a biocompatible elastomer) or agarose (a hydrogel), cell contact with rigid plastics is avoided and the mechanically tunable environment can better mimic the physiologic surrounding of the cells.

## Materials and methods

### Microwell manufacturing

Microstructured wells were made by fitting thin microstructured poly-dimethylsiloxane discs (PDMS, 15 mm in diameter) on the bottom of commercially sourced 24-well plates. The PDMS discs were replica-molded on microstructured resin molds. The positive mold surface was patterned as a densely packed hexagonal arrangement of more than 400 micro-cones of 0.35 mm of height, 0.15 mm of spacing, and 0.5 mm of base diameter. The design can be modified to obtain molds for a 6-, 12- or 96-well plate. The molds were fabricated by 3D printing using an Objet Geometries Eden 250 printer with FullCure 720 base material and FullCure 705 support material. After printing, all the produced structures were treated with a 7% NaOH for 30 min to remove any residual support material (Fig. S1). PDMS (Sylgard184^®^ 1:10 ratio of curing agent:base, DOW Chemical Company) was poured over the surface of the molds, degassed for 10 min. under vacuum, then it was covered with a glass slide to make a flat base for the device. Devices were then cured at 60 °C for at least 60 min. Devices were peeled off from the mold, and occasional excess silicone was removed with a scalpel. They were rinsed in 70% ethanol and dried under a stream of nitrogen gas. A small drop of uncured PDMS was used to fix the devices on the bottom of the multiwell plate and allowed to cure. Changing the curing agent:base ratio can enable the modulation of the flexibility of PDMS over the 0.5–4 MPa range [25]. Similar microwell devices can be made in agarose using the same 3D-printed molds. For this procedure, sterile 2% agarose (Sigma-Aldrich) in 0.9% (*w*/*v*) NaCl was poured on the positive molds and a glass slide was placed on top to provide a flat surface. After removing the master template, the agarose scaffold with highly ordered microwells was placed on the bottom of a culture well.

### Device inserts preparation and sterilization

**Fig. 1:**
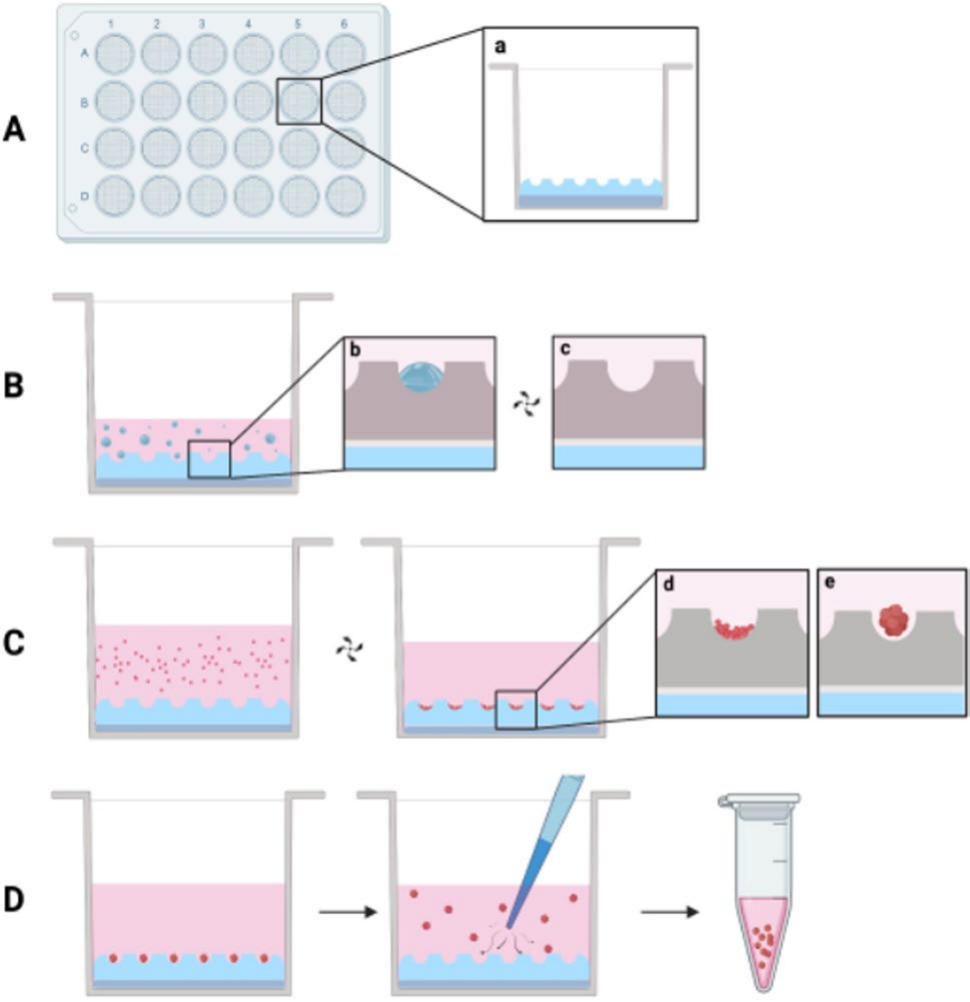
Schematic representation of prototype device workflow. **A** 24-wells plate with devices positioned on the well’s bottom (***a***) Schematic cross-section of one well showing PDMS layer for device adhesion; each device presents > 400 microwells. **B** Air bubbles trapped within microwells (***c***) air removal after centrifugation step at 70 × *g*. **C** Cell seeding and sedimentation after centrifugation, cells correctly positioned on microwells bottom (***d***) and spheroid formation afterwards (***e***). **D** Spheroids can be harvested for further procedures. Created with BioRender.com

The microwell inserts were made non-cell-adhesive by overnight incubation at room temperature with a sterile solution of 1% Polyvinyl alcohol (PVA) in water [26]. Care was taken in ensuring complete wetting of the surfaces and in getting rid of any air bubbles. Then, the wells were rinsed with PBS and sterilized with 1 mL of 70% ethanol/water for 60 min. at room temperature. The wells were then washed twice with 1 mL of sterile PBS. The wells were left overnight in PBS to ensure complete extraction of residual ethanol from the PDMS. When the plates were prepared for later use, sterile water was used instead of PBS, and the plates were then dried. Prior to use, 500 μl of cell culture medium was added to each well and the plate was kept in an incubator at 37 °C before cell seeding (see Fig. 1).

### Multicellular spheroids culture

Different cancer cell lines were tested for the generation of 3D tumor spheroids. They were obtained from the American Type Culture Collection (ATCC). HCT116 (RRID:CVCL_0291, colorectal cancer cell line); HEK293 (RRID:CVCL_0045), UOK257 (RRID:CVCL_S717, human renal carcinoma cell line); SKOV-3 (RRID:CVCL_0532, ovarian cancer cell line) were cultured in DMEM High Glucose (EuroClone, Italy) supplemented with 10% fetal bovine serum (FBS) plus 1% penicillin/streptomycin (P/S) in 5% CO_2_ at 37 °C. HRT-18 cell line (RRID:CVCL_2512, ileocecal colorectal cancer) was cultured in MEM (Gibco) 10% fetal bovine serum (FBS) plus 1% penicillin/streptomycin (P/S). TPC1 cell line (RRID:CVCL_6298, human papillary thyroid carcinoma) was cultured in RPMI medium (Gibco) 10% fetal bovine serum (FBS) plus penicillin/streptomycin (100 U/ml Penicillin G, 0.1 mg/ml Streptomycin sulfate). Neurospheres (consisting of mouse subventricular-zone isolated neural stem cells) were cultured in DMEM F12 serum-free medium (EuroClone, Italy), enriched with insulin (10 μg/mL), 1% N2, 1% B27, 20 ng/mL EGF, 20 ng/mL FGF, 2 mM glutamine and 10 units/mL penicillin and 10 ug/mL streptomycin (Sigma-Aldrich). Before cell seeding, devices were washed twice in PBS, 500 μl of the medium was then added to each well, and the plate was centrifuged at 110 × g for 5 min to remove the air bubbles trapped in the microwells. For the seeding, the desired cell concentration was diluted in 300 μl of medium and added to each well. Then, multi-well plates were centrifuged at 70×*g* for 4 min to facilitate the grouping of the cells into the microwells. For maintenance, the medium was exchanged every 48 h. During the medium exchange, care was taken not to aspirate the microaggregates by carefully pipetting on the well side without tilting the plate. The medium was added and subtracted from the same point in the well during each exchange. All cells were propagated at 37 °C in standard cell culture conditions (5% CO_2_, 95% humidity).

### Spheroid growth kinetics

Phase contrast images of tumor spheroids were taken using a Zeiss Axio Vert.A1 inverted microscope with a high-resolution camera (AxioCam MR CCD) ahead of the start of the experiments up to the end of the treatments with antitumor drugs, which commonly lasted 72 h. To measure the spheroids’ diameters, the images were examined using ImageJ software (National Institutes of Health, Bethesda, MA, USA) and the polygon tool was used to outline spheroids. The volume of spheroids was estimated with the assumption that they had a spherical shape. The same individual spheroids in a subpopulation (*n* = 28) were monitored every 24 h and the total treatment time was the same for every experiment. The difference between the volume of spheroids at the end with respect to the beginning of each treatment was used to estimate a growth rate parameter (kc). Similarly, the growth rate (k0) was computed for control spheroids that were not treated. The difference in growth rates of control spheroids (k0) against treated spheroids (kc) was divided by k0 to compute growth inhibitory effects at the end of day 3, i.e., growth inhibition = (k0 − kc)/k0. The circularity of the spheroids was measured as (4π × [Area])/[Perimeter]^2^ and ranged from 0 for the infinitely elongated polygon to 1 for a perfect circle.

### Chemicals and cell treatments

HCT116 cells were seeded into a 24-well plate with microstructured PDMS inserts (i.e., about 200 cells/microwell), following the same protocol described above. Three different compounds were tested and incubated for 72 h: Paclitaxel (Sigma-Aldrich; 25, 50, 100, 250 nM), Doxorubicin (Sigma-Aldrich; 250, 500 nM, 1, 2 μM), Suberoylanilide Hydroxamic Acid (SAHA) (Sigma-Aldrich; 500 nM, 1, 2, 5 μM). Paclitaxel and SAHA were solubilized in dimethylsulfoxide (DMSO), and the final concentration of DMSO in each cell culture was 0.1% in all cases. Doxorubicin was solubilized in distilled water. Four different concentrations of each compound were prepared by serially diluting a stock solution in cell culture media. Two wells were treated for each concentration (about 400 spheroids each) after confirmation of the homogeneity of spheroid size in each well.

### Metabolic activity assay

Cell viability was measured using an MTT (3-(4,5-dimethylthiazol-2-yl)-2,5-diphenyltetrazolium bromide) colorimetric assay. At the end of the treatments, the spheroids were harvested and resuspended in 100 μl of culture media, then transferred in a new 24-well plate and incubated with 10 μl of MTT 1 mg/mL (Sigma-Aldrich). Tetrazolium substrate was added to each well and plates were incubated for an additional 4 h at 37 °C. The spheroids were then solubilized in 800 μl of DMSO and the absorbance was measured at 570 nm using a microplate reader. The cellular metabolic activity (sometimes generically referred to as “viability”) of the treated spheroids was expressed as a percentage with respect to untreated control spheroids.

### Scaffold-based 3D culture

Cell suspensions (adjusted for seeding 200 cells/microwell) were prepared in sterile Eppendorf tubes for MCF-7 (RRID:CVCL_0031). Then, the samples were centrifuged for 6 min at 250 × *g*. The supernatant was discarded and a solution with culture media and Geltrex™ (ThermoFisher, MA, USA) at a final concentration of 2.5% was added to the cell suspension to obtain a final volume of 100 μl for seeding. 24-well plates containing the PDMS microstructured devices were previously kept at 4 °C, and a thin layer of cell culture media was added to each well. Cell suspensions were then seeded in each well and plates were centrifuged at 70 × *g* for 4 min. After 20 min at 37 °C, 500 μl of cell culture media was added to each well. Spheroid formation and diameter measurements were assessed with a light microscope and compared to spheroids generated in parallel with the usual seeding method for PDMS inserts. Images were analyzed with ImageJ software and the polygon tool was used to outline spheroids.

### Live/dead assay

Live spheroids were labeled directly while in the microstructured PDMS microwells, after removing most of the culture medium. Staining was performed with fluorescein diacetate (FDA, Sigma-Aldrich, 10 μg/mL, ex/em 494/521 nm) and propidium iodide (PI; Sigma-Aldrich, 8 μg/mL, ex/em 536/617 nm) diluted in cell culture medium without FBS. FDA labels the viable cell cytoplasm in green, and PI labels the nuclei of dead cells in red. Prior to the live and dead labeling, 150 μl of a warm 0.5% low melting temperature agarose solution was added to the microwells to avoid cell dispersion, especially in the loose peripheral layer all around the spheroids. The double staining was performed by incubation in the dark at 37 °C for 1 h before epifluorescence microscope analysis (Nikon Eclipse 80i with Hamamatsu Flash 4 sCMOS digital camera). Four different device areas were acquired with a 5X objective (Nikon, 0.15 NA) with at least 20 spheroids each, in order to analyze more than 60 spheroids per condition. Images were captured by acquiring Z-stacks of the samples using FITC (FDA) and Cy5 filter sets (propidium iodide) and merged.

### Automatic image analysis

A custom-developed software script in Matlab (MathWorks, MA, USA) was used to select each spheroid and automatically process the acquired images by image segmentation. The segmented spheroid masks were then utilized to extract the desired characteristics. In addition, a circular segmentation was performed to estimate the radial distribution of the fluorescence.

### Immunofluorescence

Spheroids cultured within PDMS microstructured inserts were washed twice with PBS for 5 min, and then, they were fixed with methanol-free 4% PFA for 20 min at room temperature, without harvesting. Samples were incubated with a blocking solution (0.3% Triton X-100 in PBS and 5% goat serum) for 1 h at room temperature. Primary antibodies (rabbit monoclonal anti-beta-actin 1:200 dilution and mouse monoclonal anti-alpha-tubulin 1:2000 dilution, Abcam, Cambridge, UK) were diluted in PBS- 0.3%Triton X-100, 1% BSA. The next day, spheroids were washed 3 times for 10 min each time with PBS-0.1% Triton X-100 and incubated with goat anti-rabbit Alexa 488-conjugated antibody 1:1000 (Abcam, Cambridge, UK) and anti-mouse Alexa-555 1:1000 for 2 h at room temperature in PBS-0.3% Triton X-100, 1% BSA. Spheroids were then washed 3 times for 10 min each time in PBS-0.1% Triton X-100, 10 min in PBS and then incubated for 10 min in Hoechst 33,258 (2 μg/mL; Sigma-Aldrich). Samples were then washed for 5 min in PBS. A 3-mm layer of 2% of low-melting-point agarose (Sigma-Aldrich) was carefully laid on the bottom of the wells to cover and embed the spheroids while maintaining their original microwell arrangement. Once firm, the agarose replicates the microstructure of the device. The spheroids are embedded at the apex of the agarose micro-cones. The agarose stub was then separated from the PDMS device and positioned face-down on a microscope coverslip. Image acquisitions of spheroids were carried out with a confocal microscope (Nikon A1-R) using either a 20X objective (NA = 0.75) or a 40X objective (NA = 0.95). Image acquisitions in the Z direction were performed using a 1 μm z-step. Automatic mosaic acquisitions of spheroids in the device array were performed.

### Phalloidin staining

Spheroids, cultured in 24-well plates within PDMS microstructured inserts, were washed twice with PBS for 5 min, and then, they were fixed with methanol-free 4% PFA for 20 min at room temperature. Samples were washed twice in PBS and incubated with a blocking solution containing 0.1% Triton X-100 in PBS for 1 h at room temperature. Spheroids were washed twice with PBS and incubated overnight with phalloidin 488 (1:800 dilution in 3% BSA- Phalloidin CruzFluor^TM^, Chemcruz^TM^). The next day, samples were washed twice in PBS and incubated overnight with Hoechst 33,342 (1:1000 dilution in 3% BSA, ThermoFisher, MA, USA). The previously described method with the agarose stub for specimen imaging was applied. For imaging, phalloidin staining of actin filaments a two-photon microscope was employed (Leica TCS SP8 X system featuring a Leica DMi8 inverted microscope) equipped with a tunable MaiTai DeepSee femtosecond laser with a tuning range of 690–1040 nm. This laser was used to excite phalloidin 488 at 976 nm using a 40X objective (HC PL IRAPO 40x/1, 10 W CORR). For each spheroid, a Z stack spanning 300 μm was acquired, collecting one frame for every 5 μm distance along the Z axis. For 3D reconstruction, the Leica Application Suite X (LAS X) software was employed. Automatic mosaic acquisitions for a large number of spheroids were performed.

### Measurements with the W8 Physical Cytometer

Live spheroids were harvested at the desired growth stage or after drug treatment. After two PBS washings, spheroids were collected in a 15 mL tube containing 1 mL of W8 analysis solution. An aliquot of the suspension was then fed into the W8 Physical Cytometer instrument (Cell Dynamics ISRL, Italy) where, for each sample, a number of spheroids were withdrawn (*n* = 24), one at a time, and subjected to cycles of controlled translation in fluidic channels. Each spheroid was analyzed twice. Numerical analysis of the captured video was then performed automatically for determining the size, mass density, and weight of each imaged spheroid. The regularity of the spheroid trajectory in the flow confirmed the sphericity of the MTS.

## Results

### Spheroids formation on prototype PDMS or agarose microstructured devices

**Fig. 2:**
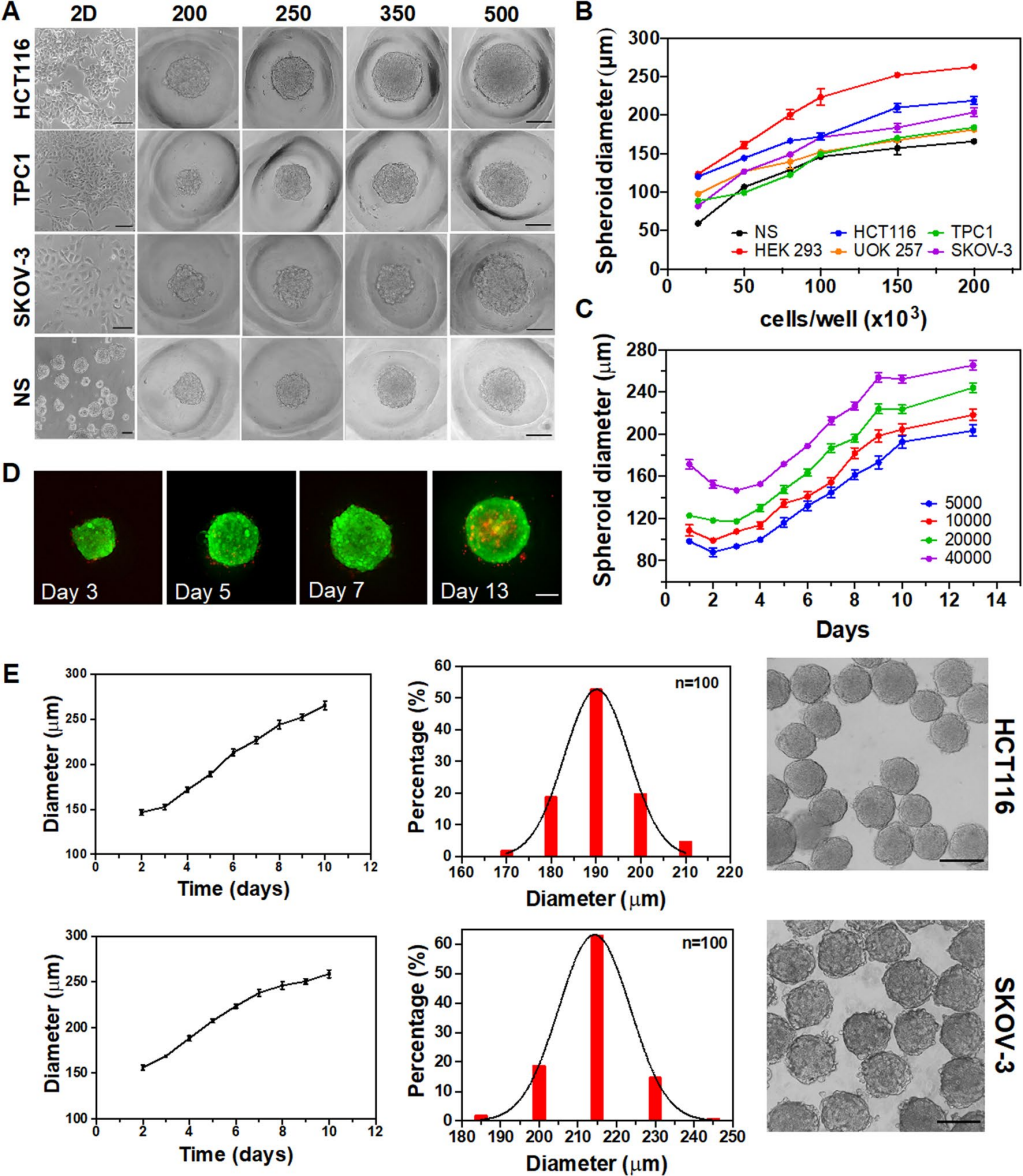
Characterization of multicellular spheroids generation. **A** Representative images of 2D cell culture and derived spheroids plated at different cell densities (200, 250, 350, 500 cells/microwell). Images were acquired 48 h post plating. Scale bar 100 µm. **B** The diameter of cell spheroids after two days of culture with a different cell seeding number (*n* ≥ 28 for each cell line). **C** HCT116 spheroid growth assay, diameter changes from different cell seeding densities. **D** Fluorescence imaging of spheroids cultured over time, live/dead staining over 13 days, scale bar: 100 µm. **E** Growth monitoring and size distribution analysis of HCT116 and SKOV-3 spheroids cultured on PDMS device. Growth curves derive from seeding 250 cells/microwell and following growth of the same spheroids over 10 days of culture (*n* = 28). Size distribution analysis was obtained from recovering spheroids cultured for 4 days. The average size of SKOV-3 and HCT116 spheroids on three different microstructured devices was 213 ± 9 µm and 190 ± 8 um respectively (*n* = 100)

In this study, attention was directed to microtumor and microtissues (non-cancer cells) formation deriving from five different human cancer cell lines (TPC1, UOK257, SKOV3, HCT116, and HRT18), one embryonic kidney cell line (HEK293), and mouse SVZ-derived neural stem cells (NS). As shown in Fig. 2A, the successful spheroid formation was obtained with different cell lines in our microdevices. It was investigated whether well-controlled tumor spheroids with long-term cell viability could be obtained from the PDMS devices. With the tested cell lines, 3D cell culture production did not require the use of any additional substance (i.e., biological matrices, synthetic hydrogels) since cells were naturally driven to adhere to each other and did not depend on matrices or scaffolds for the spheroid formation. The designed PDMS plate inserts with microwells arrays were tested as a reproducible method for uniform 3D spheroids generation. Microwell arrays allowed the generation of spheroids as well as the control of the size of the 3D cell cultures as it depends on the initial cell seeding density. Cell spheres were found to be solidly inserted into microwells and unwanted transfer of spheroids from one well to another did not occur, generally. To prove so, 500 µL of cell suspensions of the different cell types with different cell concentrations (50, 120, 200, 250, 350, and 500 cells/microwell) was seeded for growth and characterization (Fig. S2). The size of the cell clusters was shown to be strongly correlated with the initial cell seeding density, suggesting that this strategy can create size-controllable cell spheroids from a variety of cell types. It was confirmed that a higher seeding cell density produced larger cell spheres. Moreover, the size of the spheroids obtained with equivalent seeding varied according to the cell line type. As shown in Fig. 2B, at a low cell density (50 cells/microwell), HEK293 formed a larger tumor spheroid size (200 µm in diameter) than TPC1 (120 µm in diameter) and SKOV cells (150 µm in diameter). Neurospheres and SKOV-3 cell lines formed a looser aggregate compared with HCT116 or TPC1 and HRT-18 at the same cell amount. This result indicates that HCT116 was characterized by a tighter spherical structure with less ruffled borders after spheroid formation if compared with the other two cell types. Furthermore, it was observed that even a non-homogeneous three-dimensional culture such as neural stem cells can be grown in the prototype device giving uniformity and control in the size of this type of cell culture type. To validate this technology for long-term monitoring of spheroids, the growth of HCT116 spheroids with different cell seeding concentrations (10, 20, 40, 100 cells/microwell) was tracked from day 1 post-seeding up to 13 days (see Fig. 2C).

Several stages of morphological changes occurred during spheroid development. Individual cells could be easily identified in the early stages when they layer the microwells in a quasi-2D fashion. Then, cells spontaneously self-assembled to create cell aggregates in each microwell. Cell aggregates then began to merge and morph into a single sphere in each microwell, as a result of intercellular interactions and connections. After that, multicellular spheroids developed as solid structures with a smooth and continuous surface. The spheroid borders became ruffled in the final stage, indicating cell proliferation at the spheroid periphery and individual cells were no longer recognizable. The process of spheroid formation after seeding is presented in Supplementary Video 1 (for a device portion of 50 microwells). The mean apparent MTS diameter of all cell types increased over time, after an initial slight decrease due to the 3D compaction of a wider arrangement of quasi-2D cells (see Fig. 2C). Thereafter, around day 9, the diameter reached a plateau. The growth rate of spheroids with different sizes (10, 20, 40, and 100 cells/microwell) was almost independent of cell seeding concentration, about 46%/day. Live/dead staining analysis in Fig. 2D shows a high cell viability of the spheroids over 13 days of culture.

The PDMS microwells were developed to accommodate enough cells while also protecting the formed spheroids from direct exposure to the fluid flow during the medium exchange. Unprotected media exchange on spheroids culture could cause the shedding of PI-stained dead cells from the outer layer of the spheroids. Furthermore, the microfabricated devices allowed us to trace the same spheroids over time by microscopy inspection, thus allowing also to define the individual spheroid growth for different cell lines. Very homogeneously sized spheroids were obtained: 97% of the HCT116 spheroids had a diameter between 180 and 210 µm at the end of the time of growth, while 99% of SKOV-3 spheroids were between 185 and 230 µm. As shown in Fig. 2E, after the harvesting procedure, the spheroids shape and compactness were not altered. The results demonstrate the proper formation and facile monitoring of large sets of homogenous spheroids: such results can be more challenging to achieve using different existing methodologies, such as purely scaffold-based techniques (Fig. S3).

The same 3D-printed resin molds used for generating PDMS microfabricated devices could be used also for agarose device production without any adaptation. Spheroid production capacity and uniformity provided by each material were compared. As shown in Fig. S4, cell spheroids were successfully produced with both materials, and no significant differences in spheroids growth behavior were encountered between agarose and PDMS inserts during our experiments (Fig. S4). Spheroids growth was analyzed on three different devices for 5 days of culture, showing that agarose and PDMS devices produced cell spheres with comparably good uniformity in size and shape.

The morphology characteristics and growth/viability of spheroids formed using two different commercial systems were evaluated in a comparison with our proposed prototype devices. Elplasia^®^ 24-wells plate with round bottom wells (Corning, Life Science) and Spherical plate 5D 24-well plate (Kugelmeiers^®^) were used. The growth rates of the spheroids were within 20% of each other in the three platforms (see Fig. S5).

### Use of biological matrices for spheroids generation in the prototype microstructured PDMS devices

Our previous results showed successful formation and growth of different cell lines in the fabricated prototype device. Without the use of a supporting scaffold, spheroids were created by letting cells to self-assemble into clusters and grow. Another common approach in 3D culture is to produce cell aggregates through matrix-assisted procedures. 3D scaffolds can be made of biological or synthetic polymers and generally necessitate a precisely controlled environment in terms of temperature and pH [27–29]. In order to grow in 3D, certain cell lines require components in their growth media that imitate the extracellular matrix (ECM), such as laminin or collagen IV. These components are important for cell/extracellular matrix interactions and can provide an environment conducive to the development of intercellular connections [30, 31]. For the development of 3D compact cell clusters with MCF-7 breast cancer cells, the use of an extracellular matrix was investigated. In fact, the use of the PDMS device without matrix led to loose and not uniform spheroids from this cell line: after a few days of culture, multiple MCF7 spheroids were found in each well and these generally had an irregular and heterogeneous morphology (Fig. S6A). In order to improve the effectiveness of the proposed technology and adapt it to this type of cell line requiring a matrix, the microwell structures were combined with the use of a scaffold. For these experiments, Geltrex™ LDEV-Free (Reduced Growth Factor Basement Membrane Matrix, Gibco^®^) was used as an extracellular matrix. MCF-7 cells were seeded at about 250 cells/microwell in the PDMS prototype inserts. Then, several Geltrex™ concentrations and conditioning regimens were tested (i.e., addition just after cell seeding or 24 h after cell seeding; concentrations of 9 mg/mL to 15 mg/mL). The results showed that when Geltrex™ was added during cell seeding, tightly packed spheroids were detected in the presence of 10–12 mg/mL of Geltrex™. This procedure allowed us to obtain homogeneous and compact spheroid formation. Spheroids were monitored from day 1 to day 7: growth curves showed a daily volume growth rate of 73% for MCF-7 (Fig. S6B *N* = 28). MCF-7 spheroids circularity, calculated from 2D projection images, varied significantly according to the presence or absence of Geltrex™ In fact, the use of matrix provided regular cell clusters with the circularity of 0.90 ± 0.02. On the contrary, spheroids generated with the standard procedure were characterized by circularity of 0.6 ± 0.1 (Fig. S6C).

### Characterization of drug response of MTS in microwells

As a proof-of-concept of the relevance of the presented microwells-based microsystem, the effect of cytotoxic drugs on spheroids was investigated on the widely used colorectal cancer cell lines HCT116. Due to their effectiveness and widespread use in clinical therapies, Paclitaxel and Doxorubicin were employed as model compounds to assess the potential usefulness of this prototype platform for anticancer drug screening. Vorinostat (SAHA) was additionally included due to its promising anticancer activity on this type of cancer: this compound significantly inhibits the expression of HDAC proteins in colon adenocarcinoma cells and in tumors of nude mice providing a possible effective treatment for patients [32, 33].

**Fig. 3:**
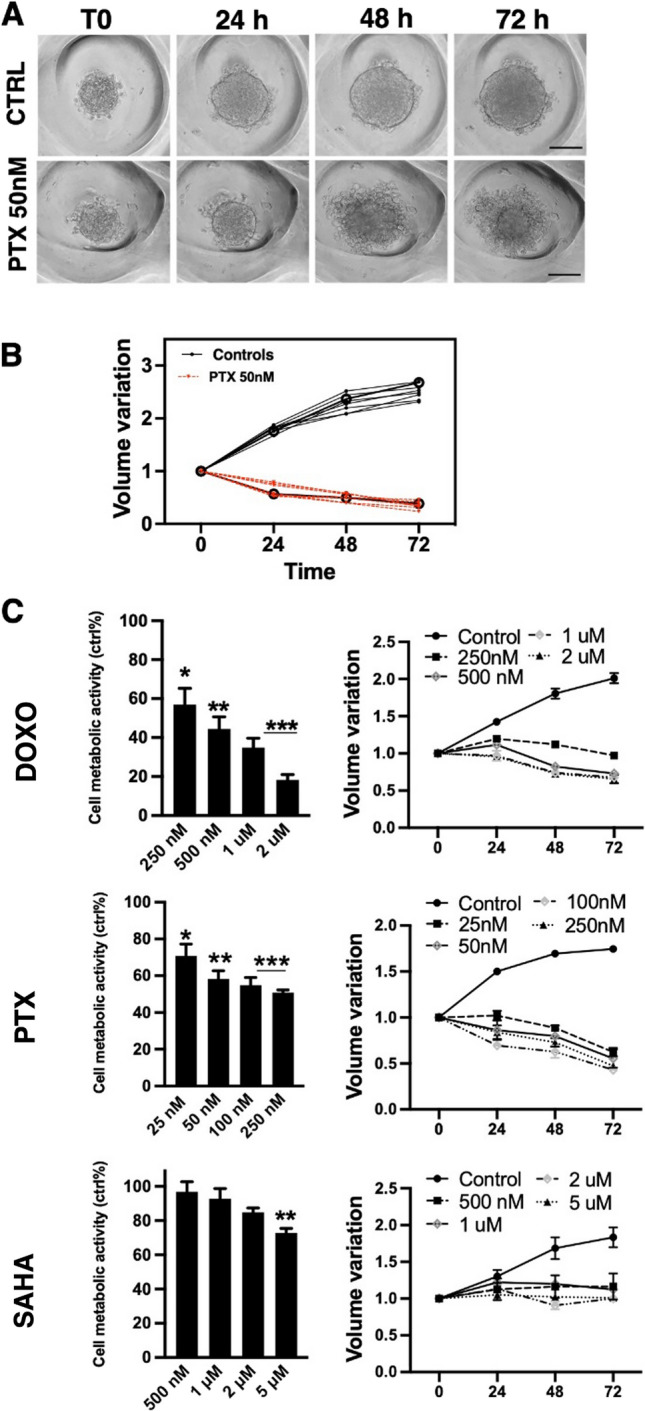
Compounds effect on spheroid growth, morphology, and cell metabolic activity. **A** Representative brightfield images of one control and one treated HCT116 spheroids in a PDMS device. Images were used for generating growth curves, tracking the same sets of spheroids over time. Scale bar: 100 µm. **B** Volume variations of individual control and treated spheroids relative to a device area monitored over time (*n* = 7 each). Thicker lines with round markers represent the average growth curves of treated and control samples. **C** Analysis of the metabolic activity (with MTT assay) and growth curves of spheroids treated with DOXO, PTX, and SAHA. Samples were evaluated in triplicate. Each data point indicates mean ± SEM (Growth curves represent *n* = 28 per condition, Student’s t-test indicated that this result was statistically significant; *P < 0.05, **P < 0.01, ***P < 0.001 versus control)

The effect of Paclitaxel, Doxorubicin, and SAHA on preformed HCT116 spheroids was examined. A 4-days initiation interval for spheroid formation was found to reproducibly create spheroids of 160–200 µm: this starting spheroid dimension is usually employed at the onset of treatment for drug testing [34]. Different concentrations of the compounds in the growth media were used to treat the spheroids for 72 h. Measurements of the core spheroids volume (estimated from their equivalent diameter) were performed at 0 h, 24 h, 48 h, and 72 h (Fig. 3A). Averaged data representative of the spheroids volume variations is shown in Fig. 3B and C (*N* = 28 spheroids tracked, per condition). The MTT cytotoxicity analysis was additionally performed as an end-point assay after each treatment to determine the drug effect on tumor spheroids. It showed that all drugs suppressed cancer cell growth.

In Paclitaxel-treated microtumors, as shown in Fig. 3C, a slight inhibition was observed for concentrations > 25 nM whereas concentrations between 50 and 250 nM showed similar viability reductions of around 50%. This compound also caused a peculiar effect on the HCT116 spheroids: these acquired a looser appearance with cells gradually detached at the edges. Microtumors exhibited an evident decrease in size and spread with Paclitaxel. Spheroids presented an outer layer of detached cells, and the compact central core was identified and measured for volume analysis during treatments. Untreated spheroids doubled their volume while treated ones showed a growth inhibition of up to 180% after 72 h of treatment with the highest dose of 250 nM.

Doxorubicin treatment caused a dose-dependent reduced metabolic activity of HCT116 spheroids indicating that the drug significantly induced cell death in the colon cancer cell line. The growth curves of the spheroids treated with 1 μM and 2 μM showed similarity: after 72 h the growth inhibition was only 5% higher in 2 μM compared to the 1 μM drug (125% and 120% respectively).

SAHA showed significant viability reduction at concentrations > 1 μM. With respect to the controls, the cores of treated spheroids showed no size increase remaining at the initial volume. Growth inhibition after 72 h for the maximum concentration dose of 5 μM was 99%. 

The MTT assay was performed after drug treatments on MTS of various sizes, and the results expectedly confirmed a negative correlation between the size of the tumor spheroids and the sensitivity to the treatment (Fig. S7).

Additionally, it was investigated if the sensitivity of spheroids for Paclitaxel varied among the different microwell culture systems (our prototype devices *vs.* the two commercially tested systems, vide supra). The metabolic activity of spheroids treated with 25 nM PTX was measured using the MTT assay and normalized to control untreated spheroids generated on the same platform. The PTX treatment reduced the viability of about 20% in all three spheroids populations obtained in the three microwell systems with no significant differences among them (Fig. S5).

The cytotoxicity tests performed on MTS in our microwells devices report a reduced sensitivity to drugs with respect to the same cell lines exposed to the same drugs while cultured in 2D, as also expected from literature data. The half maximal inhibitory concentrations for 3D MTS in our microwells (IC_50_) were about 280 ± 80 (S.E.M.), 38 ± 3 and 1600 ± 600 nM for Doxorubicin, Paclitaxel, and SAHA, respectively (see Fig. 3C). These were 215%, 122%, and more than 300% of the values found by us or reported in the literature for the same drugs and cell types in 2D culture [35, 36]. Cytotoxic drug effects in spheroids cultures with comparison to monolayer and the determination of IC_50_ are reported in Fig. S8.

In order to further characterize the MTS, we produced and the effects of drugs on them, we employed a recently-introduced physical cytometer (W8, Cell Dynamics, Italy) [36, 37]. This analysis could determine the individual diameter, mass, and density of a small population of MTS (*n* = 24). The analyzed spheroids turned out to have very homogeneous diameters, mass, and densities, thus confirming not only the narrow size distribution of the cultured MTS, but the homogeneity of their cellular density (Fig. S9). A significant difference was expectedly found after PTX treatment (reduction in spheroid size and density, broadening of the distributions).

### Live/dead assay performed in the microwells

Live/dead staining visualizes the distribution of live and dead cells in the MTS and can correlate with the metabolic activity assay. It can give information on the mode and kinetics of the action of cytotoxic drugs. The presented microwell technology allows for the performance of live/dead fluorescence staining in situ (in the devices), so that the derived structural data could be also matched with the characterization and growth curves of individual spheroids. Spheroids of the HCT116 cell line were exposed to Paclitaxel for 72 h, and the fluorescence signal intensity and spheroids morphology were imaged directly in the microwells device and processed with a custom-developed semi-automated image-based segmentation analysis. Spheroids were not harvested prior to measurement also to avoid losing information about the possibly fragile outer layer of dead cells. Live spheroids cultured in the PDMS devices were directly stained with fluorescein diacetate and with propidium iodide for identifying live and apoptotic cells, respectively. In order to preserve most of the cells of the outer diffuse layer, the device surface was gently embedded in a thin layer of low-melting-point agarose before staining the cells (see “Materials and methods”). This method allowed easier handling during and after staining. Thanks to this procedure, the microwell PDMS plate inserts can be extracted from the plate wells without disrupting the MTS. The thin layer of agarose allows face-up or face-down specimen observation with an upright (Figs. 2D and 3A) or an inverted microscope.

**Fig. 4:**
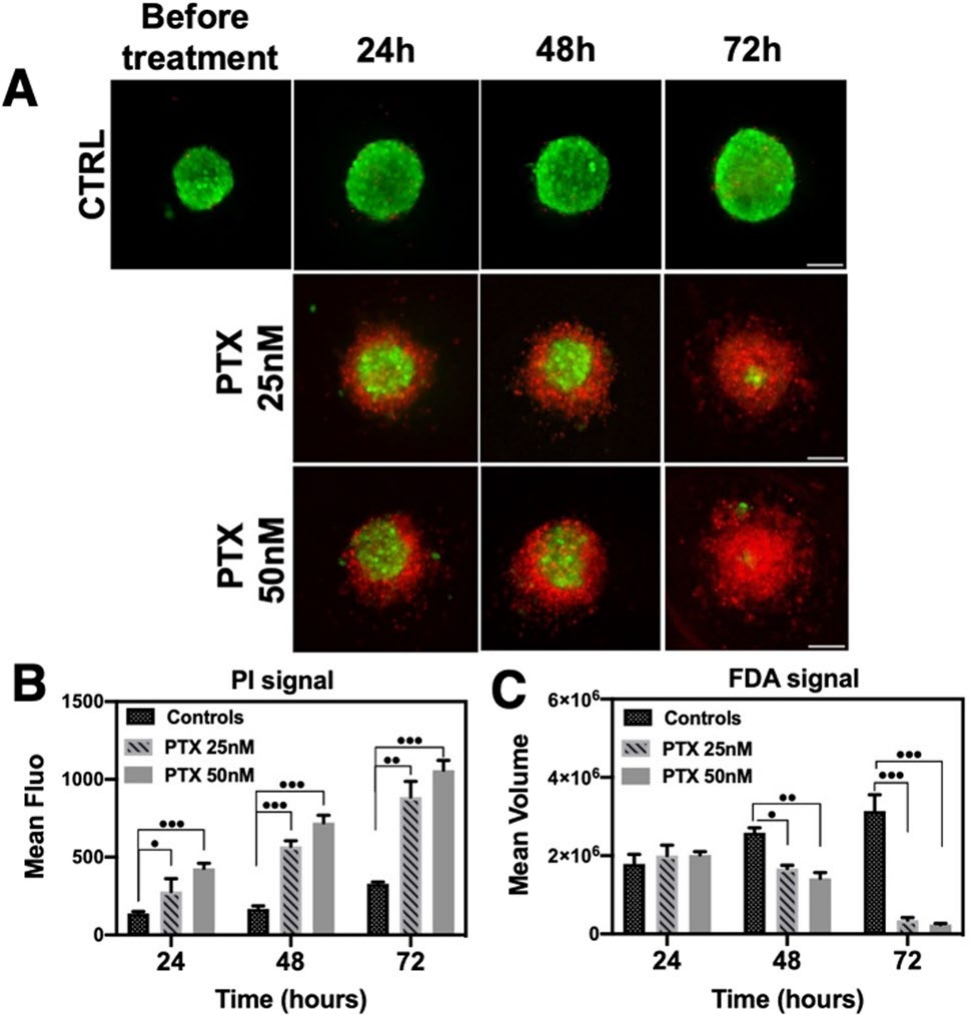
Live/dead staining images of drugs treated HCT116 spheroids. **A** Spheroids were treated with different concentrations of PTX for 24 h, 48 h, and 72 h and then stained with FDA and PI. The fluorescence levels of each channel were mapped to the same look-up tables for the three conditions. **B** Normalized mean fluorescence intensity relative to propidium iodide signal. **C** Normalized mean volume calculated from FDA-signal segmentation. Bars represent the averaged data of > 60 spheroids per condition, Student’s *t*-test, treated versus control at each time point; *P < 0.05, **P < 0.01, ***P < 0.001 Error bars indicate SD. More detailed images can be found in Fig. S10

As shown in Fig. 4A, during the first 24 h, spheroids treated with 25 nM of PTX showed irregular shapes. At the highest concentration of 50 nM, the increase of dead cells (PI +) was detected. After additional 24 h, a diffuse outer layer of dead cells appeared in both conditions. Finally, after 72 h, only a central viable core was detected, with a pronounced increase in levels of dead and apoptotic cells in the peripheral layer of the spheroid. Control untreated spheroids did not present any diffuse layer and did not change in shape.

Figure 4B shows the mean relative intensity of fluorescence of PI + cells of more than 60 spheroids per condition. First, a dose-dependent increase in PI fluorescence intensity was observed over time. This response most closely matched those measured with the MTT assay, validating such results (see Fig. 3). Moreover, it can be appreciated that the volume of the FDA + core gradually decreases as a function of the PTX concentration (Fig. 4C). Live/dead analysis provided complementary structural information on the drug-induced apoptosis within the spheroids, making it possible to describe the mode of action of the drug and to validate the MTT data.

### In situ fluorescence analysis performed in the device

Using the presented PDMS devices, all the experimental steps of immunostaining procedures (i.e., PBS rinsing, medium exchange, sample fixation, and antibody incubation) can be performed in the same multi-well plate with no spheroid manipulation, resulting in the treatment and labeling of multiple spheroids at the same time. Samples could be imaged with high-resolution optical microscopy such as confocal fluorescence microscopy and two-photon microscopy. Devices can be easily removed from support plates if needed. As explained above, a thin layer of agarose can enable the extraction of the PDMS multiwell devices without disrupting the arrangement and structure of MTS to allow for large field-of-view imaging with long working distance objectives.

Alternatively, by means of the agarose replica technique (*see Materials and Methods*), spheroids can be trapped at the apex of agarose cones and then imaged at high resolution maintaining the microwells array and using short working distance, high numerical aperture objectives in confocal microscopy. As an example, immunofluorescence of cytoskeletal proteins has been performed and a device area including 14 spheroids has been acquired at high resolution (Fig. S11).

**Fig. 5:**
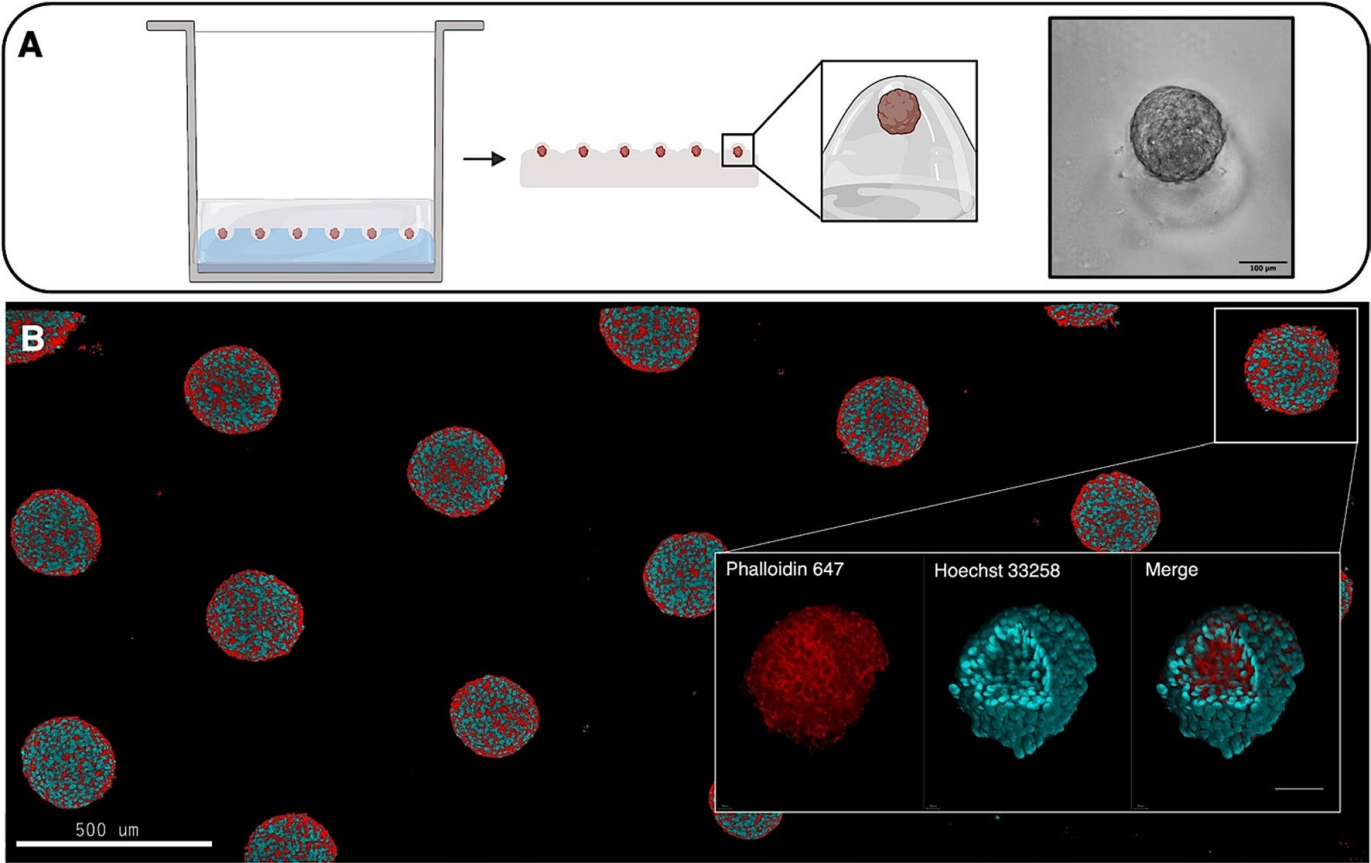
A transfer method allows collecting a high number of spheroids to perform image analysis while maintaining the MTS positions in the array. **A** Illustration depicting the method for spheroids embedding and harvesting. **B** Two-photon images of spheroids cultured in prototype devices. The image in the background was obtained by tiling confocal scans of HCT116 spheroids embedded in agarose (40X magnification, scale bar: 500 μm). The phalloidin 488 emission is in red (to maximize contrast) while the blue emission is from Hoechst 33,258. The inset image at the bottom represents a 3D reconstruction of a single spheroid (Scale bar: 100 μm)

The full structure of many spheroids can be characterized with this method using a suitable microscopy technique, maintaining a correlation with the growth data of the individual spheroids. Figure 5B shows a tiled two-photon confocal microscopy image covering more than 21 spheroids within the same focal plane, imaged in 3D at high resolution. This gives access to high-throughput characterization of 3D spheroids, an added value of the proposed system in consideration that other state-of-the-art methods commonly require sample transfer to specific microscopy plates for high-resolution imaging [38, 39]. Spheroids collection and the numerous washing and centrifugations required for standard immunostaining protocols may lead to sample loss, spheroids fusion, and damage. If desired, low-melting-point agarose used for spheroid immobilization or for the replica can be subsequently melted to recover the previously stained spheroids for further molecular characterizations.

## Discussion

As 3D cell culture is nowadays recognized as an advancement in recapitulating the behavior of cells in vivo, especially in making cell models of cancer and testing therapeutical approaches, a new abundance of culturing methods and devices have flourished recently. The current trend valorizes the methods for high throughput screening (HTS) that can be simple to use, customizable, and potentially adaptable to several cell growth conditions and characterization procedures. Automation in cell culture and analysis of large sets of samples (sometimes termed High Content Screening, HCS) also calls for standardization and parallelization of culture devices. The available procedures aim, for example, at producing large amounts of multicellular tumor spheroids (MTS), some of the simplest 3D models of cancer, while more complex models could also be of interest. While MTS can be successfully grown by seeding cells in a hydrogel matrix of biological origin (scaffold) that mimics the extracellular matrix, the result is generally a highly heterogeneous distribution of MTS where individual cells, small cell aggregates, and larger spheroids are found in the same volume (see an example image in Fig. S3), making it difficult to study their growth and their response to stimuli, such as drugs. MTS of different sizes grow differently and respond differently to drugs and other environmental changes, as they might be made of different cells with different behavior (active, quiescent or hypoxic, dead) even when made from a single cell line and cells could be shielded from the environment and exchanges of gases, nutrients, and metabolites by cell layers of different thickness, or different amounts of extracellular matrix. Consequently, numerous efforts have been directed into making cell culture methods that can produce a large number of homogeneous MTS (with the same size and morphology). While the classic “hanging drops” method could represent a possible solution, more robust, flexible, and easier-to-use methods are taking the stage. Several examples of microstructured culture devices are available commercially or have been presented in research papers, recently. Concurrently, 3D bioprinting also proved as a viable technique to form individual MTS. For example, classical or mask-free photolithography has been used to pattern surfaces into vast arrays of small cavities to host cells [17, 40, 41]. While effective, such fabrication technologies are difficult to source and expensive. Furthermore, photolithography produces mainly flat-bottomed cavities to host cells and the resulting spheroids, while rather homogeneous in size, turn out to be heterogeneous in shape and apparently not engaging all possible cell-to-cell contacts. Self-patterning surfaces have also been proposed for MTS culture [42] and, while interesting, they do not provide a direct method to modify the pattern, the shape, and the size of the cavities where cells are grown and the necessary materials could pose problems of biocompatibility. 3D bioprinting is undergoing furious development nowadays and it will certainly provide valuable methods to produce MTS. Currently, the achieved quality needs improvement, printing is time-consuming and the MTS less homogeneous [13, 43, 44] and in lower numbers than desired [44]. Additionally, bioprinters are still a rather expensive and complicated piece of equipment for the cell biology lab. In our opinion, additive 3D printing or CNC micromachining techniques are currently still the best available solution to make molds for producing arrays of cavities (microwells) for MTS growth: large arrays of MTS can be easily produced for HTS, and this technique is available to many users [46, 47]. While commercially available devices commonly employ hard plastics that might be undesirable, albeit effective (see the Results section for commercial names), some research applications explored the use of elastomers [40] or hydrogels [48].

As we have shown in the Results section, we herein presented a method to make elastomeric or hydrogel culture devices that offer large arrays of cell culture microwells of defined shape to grow and host spherical MTS. The devices have been made using 3D-printed resin molds with the desired, tunable geometry and arrangement. Molds can be reused numerous times, making the process especially inexpensive. The individual features of the molds are about half a millimeter in size, and they can be manufactured with precision with high-end photolithographic additive 3D printers, which is nowadays relatively common. Our proposed devices, produced with such molds, are designed to be inserted in standard multi-well plates, enabling familiar handling procedures and fitting in automated live-imaging and HCS instruments (thin PDMS is optically transparent and allows the direct inspection of the MTS growth). With respect to other examples in the literature [48], we could exploit additive 3D printing to make smaller features and obtain culture devices of different materials.

It is common practice while using commercial plates or other prototype devices to harvest and transfer spheroids in new containers after drug treatments [49], to perform imaging or immunofluorescence [50–52]. As this complicates handling and data acquisition, our efforts were directed towards developing devices and dedicated protocols that allowed to perform many in situ characterizations, including immunofluorescence, without the need to harvest the MTS from the culture microwells. MTS can optionally be transferred from the culture devices for further growth or characterization, either keeping them in the elastomeric devices or harvesting and disrupting their ordered arrangement. As long as the MTS are arrayed in the culture device, individual spheroids can be traced in their growth or response to drug treatments (as shown in Fig. 3B) providing HCS variability of the biological behavior of the spheroids. After the desired culture time, each PDMS microwell device can even be removed from the plate well, if desired, for off-plate characterizations (such as confocal microscopy) or to recycle the device after cleaning and sterilization. The close arrangement of microwells in the plane allows facile high-resolution microscopy observations of large numbers of spheroids (see Figs. 5**, **S10, S11, and Video S1 for examples).

Very homogeneous populations of MTS were obtained with our devices, analogously or sometimes better than with other presented microwell systems. Size homogeneity is intrinsic in the microwell strategy and is guaranteed by the arrangement of microwells that split the cell seeding evenly and losslessly within the plate well. The control of spheroid morphology is obtained thanks to the round-tip conical shape of the cavities that seconds the natural tendencies of many types of cells aggregates to form spheres. These features can be tuned on purpose for different cultural conditions through dedicated 3D printing of the reusable molds. In Table 1, a comparison of the different techniques for producing MTS is presented. Our proposed culturing devices and protocols perform with intra-device homogeneity and inter-experiment reproducibility in line with the best examples in the literature and with commercial microwell plates (see “Spheroids formation on prototype PDMS or agarose microstructured devices” and Fig. S5).

**Table 1:** Quantitative comparison between our method and other methods

Method	MTS mature morphology and growth timing	# Cell Required	Diameter (µm) (Range, mean ± SD, CV%, n)	Amount of collected spheroids	Fluorescence analysis (Procedure, image quality)	References
Hanging drop	3–7	250–3000/drop	200–600, 359 ± 95 38.5 µm, 26.5%, 34	1/drop	MTS recovery, low quality	[9–11]
Ultra-low attachment plates	3–7	5 – 20 (× 10^3^)	300–600, 312 ± 23, 7.37%, 30	1/well	MTS recovery, high quality	[12, 53]
Scaffold embedded MTS	7–10	1000–10,000/drop	15–350, 96 ± 55, 58%, 300	Numerous, variable	MTS recovery, low quality	**
Hydrogel 3D Bioprinting	3–6	~ 4700 cells/drop	200–500, 190 ± 13, 4.2–8.7%, 6	1/well	MTS recovery, high quality	[44]
	5	700 cells/well	﻿Average area: 1480 μm^2^, n/s, n/s, CV:17%, n/s	Numerous	Low quality	[13]
	7	﻿5 × 10^4^ cells/scaffold	100–500 µm, 410 µm, n/s, 3 ﻿	Numerous	Low quality	[45]
This method	*1–15	10–100 cells/microwell	50–300 µm, 190 ± 8, 4.8–6.2%, 100	> 400/well	In situ staining, immunofluorescence, very high quality	

*SD* standard deviation, *CV* coefficient of variation, *n* number of spheroids analyzed, *n/s* not specified

*Depending on which size spheroid was required, larger ones needed more time

**Scaffold-embedded spheroids were obtained using Geltex™, numerical data refer to an initial seeding density of 10.000 cells/15 µl drop (Fig. S3)

It is becoming increasingly accepted that the mechanical properties of the extracellular matrix alter the properties and phenotype of cells [22, 23]. Cancer cells with different spreading abilities often possess a different mechanical rigidity [54]. Also, the rigidity of the extracellular matrix can be itself modulated by the activity of cells and cells can adapt differently to matrices of different rigidity [55]. It has been recognized that the high rigidity of culture plastics could be limited in recapitulating the mechanics of living tissues and it might impose unnatural growth conditions on the cells [23]. The culture approach presented in this work allows fine-tuning of the mechanical properties of the culture environment as several different materials can be used for fabricating microwell devices. PDMS allows for tuning the rigidity of the material in the MPa range (as described by Young’s modulus), about three orders of magnitude softer than culture plastics [25]. Agarose allows to tune the rigidity in the kPa range, instead, close to the physiological rigidity of many tissues [56]. The rigidity of the materials can be tuned by changing the crosslinker ratio (for PDMS) or the gel concentration (for agarose). The effects of such tunability on the cells were not yet fully tested in this work. Additionally, it is expected that other types of hydrogels could be replica-molded for agarose and PDMS to make microwells. This could provide a further choice of conditions that could match the mechanical properties of the culture environment with that of the growing spheroids or those of the host tissues where the researcher wants to test the spheroid growth. Materials with different permeability to gases can additionally impose different cultural conditions. Analogously, it could also be possible to achieve gradients of growth factors to simulate complex physiological conditions by preparing multi-layer hydrogels.

Our results indicated that PDMS devices allowed easy and fast spheroid formation. This platform supported media volumes of up to 500 µL (in the 24-well plate format), enabling the growth of spheroids without significant media depletion over 13 days. Furthermore, the present work investigated system adaptability for spheroids generation with microwell devices made of different materials (PDMS and agarose) and growth conditions (scaffold-free and scaffold-based culture). For example, Geltrex™ was employed for the development of 3D compact spheroids from the MCF-7 cell line. In fact, in our hands and in the literature, methods using Geltrex™ alone for 3D culture led to the formation of extremely heterogeneous MTS (Fig. S3), while bioprinting can be used to seed spheroids in biological matrices, albeit with the mentioned complexities. As MTS uniformity is a value, we believe that our methods and devices could represent a good solution for the use of matrices/scaffolds and the simple obtaining of homogeneous MTS.

Various HTS technologies employing microstructured-based surfaces have been designed to determine treatment responses in cancer [40, 57, 58]. Our fully in situ growth of more than 400 spheroids per multiple well increased the efficiency of size control and made sure that all spheroids were situated in the same plane close to the plate bottom, which facilitated growth monitoring by imaging (and HCS). The peculiar properties of the PDMS devices allowed for performing many in situ characterization techniques allowing the users to still track the properties of individual MTS along their growth and response to treatments. Fabricating the devices in agarose or other hydrogels further extends the possibilities to closely mimic the cell growth environment.

## Conclusions

A method for producing microstructured devices was presented that proved suitable to generate hundreds of homogenous spheroids from different cell lines. These were fully compatible with commercial multi-well plates and could be made of PDMS or agarose. 3D multicellular spheroids could be cultured with or without scaffold matrices yielding a large number of highly homogeneous spheroids. Drug response could be successfully analyzed in a quantitative manner, and simple methods for in situ fluorescence analysis could be developed, as examples of possible spheroid characterizations. This model may be a promising approach not only for drug screening applications, but also for other high-throughput spheroids applications. The presented method should prove useful for preclinical screening and assessment of anticancer drugs for a variety of tumors. As the devices can fit into standard multiwell plates, they can be used within commercially available high-content screening platforms and it is conceivable that small upgrades in the software of such instruments could lead to the facile automated analysis of large numbers of individual spheroids tested in parallel in many conditions. In future studies, MTS could be obtained with these devices from co-cultures with other relevant tumor microenvironment cells or non-malignant to develop a more complex 3D tumor model for even better modeling of solid tumors and their microenvironment.

### Supplementary Information

Below is the link to the electronic supplementary material.

Supplementary file1 (PDF 1420 KB)

Supplementary file2 (MP4 2148 KB)

## Data Availability

Supplementary data associated with this article include the results obtained with the prototype devices with additional cell lines, results obtained using agarose devices, comparison with commercial culture plates and other 3D cell culture methods, characterization of spheroids physical properties, and a short time-lapse video of MTS generation.

## Abbreviations

PDMS: Polydimethylsiloxane
MTS: Multicellular tumor spheroid
HCS: High-content screening
HTS: High-throughput screening
PVA: Polyvinyl alcohol
NS: Neurospheres
SAHA: Suberanilohydroxamic acid
PTX: Paclitaxel
DOX: Doxorubicin
FDA: Fluorescein diacetate
PI: Propidium iodide
PBS: Phosphate Buffer Solution
