# Supplementary material for: Microstructured soft devices for the growth and analysis of populations of homogenous multicellular tumor spheroids
Source: Cell Mol Life Sci. 2023 Mar 16;80(4):93. doi: 10.1007/s00018-023-04748-1 (PMC10020259; doi:10.1007/s00018-023-04748-1)
Supplement: Supplementary file 1 — Supplementary file1 (PDF 1420 KB) [file 18_2023_4748_MOESM1_ESM.pdf]

## Supplementary Information for:

# Microstructured soft devices for the growth and analysis of populations of homogenous multicellular tumor spheroids

*Cellular and Molecular Life Sciences*

Ottavia Tartagni[1] | Alexandra Borók[2] | Emanuela Mensà[1] | Attila Bonyár[2] | Barbara Monti[1,3] | Johan Hofkens[4] | Anna Maria Porcelli[1,3] | Giampaolo Zuccheri[1,3,5*]

*[1] Department of Pharmacy and Biotechnology, University of Bologna, Via San Donato, 19/2 - Bologna, Italy, 40127;*

*[2] Department of Electronics Technology, Budapest University of Technology and Economics, Budapest, Hungary;*

*[3]Interdepartmental Center for Industrial Research on Health Sciences & Technologies at the University of Bologna*

*[4]Department of Chemistry, KU Leuven, Leuven 3001, Belgium, and [5]S3 Center, Institute of Nanoscience, Italian National Research Council*

*Corresponding author: giampaolo.zuccheri@unibo.it

**S1: design and realization of 3D printed molds**

3D renders were designed with SolidWorks (2013) CAD software (https://www.solidworks.com/). Further modifications have been made regarding cones geometry. Before selecting the final mold for devices production various mold designs were used to test spheroid generation with different cell lines.

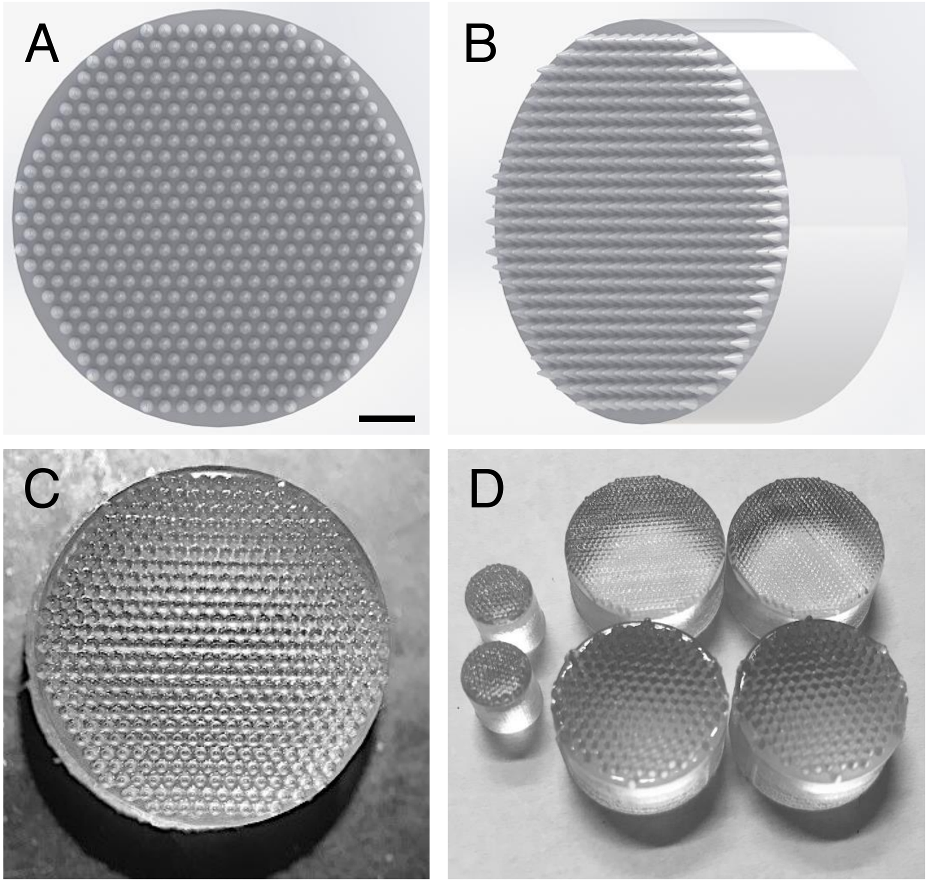


**Fig.S1: Master molds fabrication design.** (A, B) 3D render of master mold front and side view, details of cones array on mold surface (scale bar: 2 mm). (C) Master mold chosen for device production. (D) Master mold with different dimensions (for different multiwell plate types) and cones arrangement made with 3D printing technique.

## S2: Culture of additional cell lines in the microstructured elastomeric devices

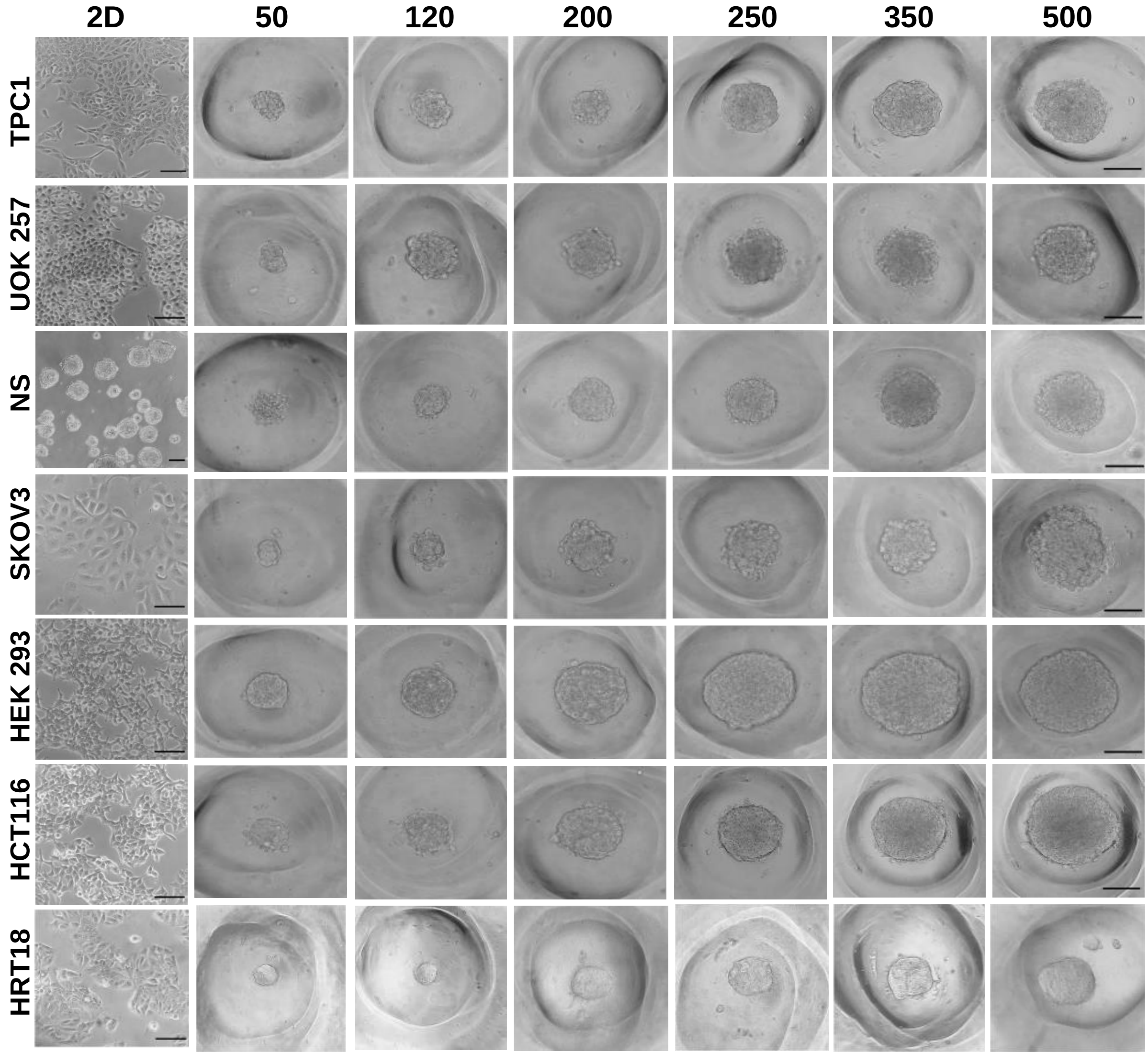


**Fig.S2: Different cell lines tested on the prototype microwells device: 2D morphology and relative 3D culture.** Spheroids of seven cells lines seeded at different cell densities (50, 120, 200, 250, 350 and 500 cells/microwell). Images of cellular aggregation and morphology were acquired under bright field microscopy after 48h of culture. Scale bars: 100 µm.

## S3: Comparison to different spheroid creation method

Geltrex$^{TM}$ was employed as extracellular matrix proteins. HCT116 cells were seeded at 1000, 2000, 4000, 10000 cells per well in flat-bottom 96 well plates. The different seeding concentrations were tested to be comparable with the number of cells used for experiments with the prototype device. Area, diameter, and circularity analysis of the spheroids generated with scaffold-based technique was performed. From these results, it is possible to observe a marked heterogeneity of the MTS. The 3D culture showed spheroids merging with each other over time. The graphs show that the average area and diameter of the spheroids increase according to the cell seeding density, but the variability of the dimensions is extremely high when compared to the microwell method. Furthermore, the formation of spheroids at different focal planes complicates the morphological analysis, also making it difficult to track the growth of the same spheroids over time.

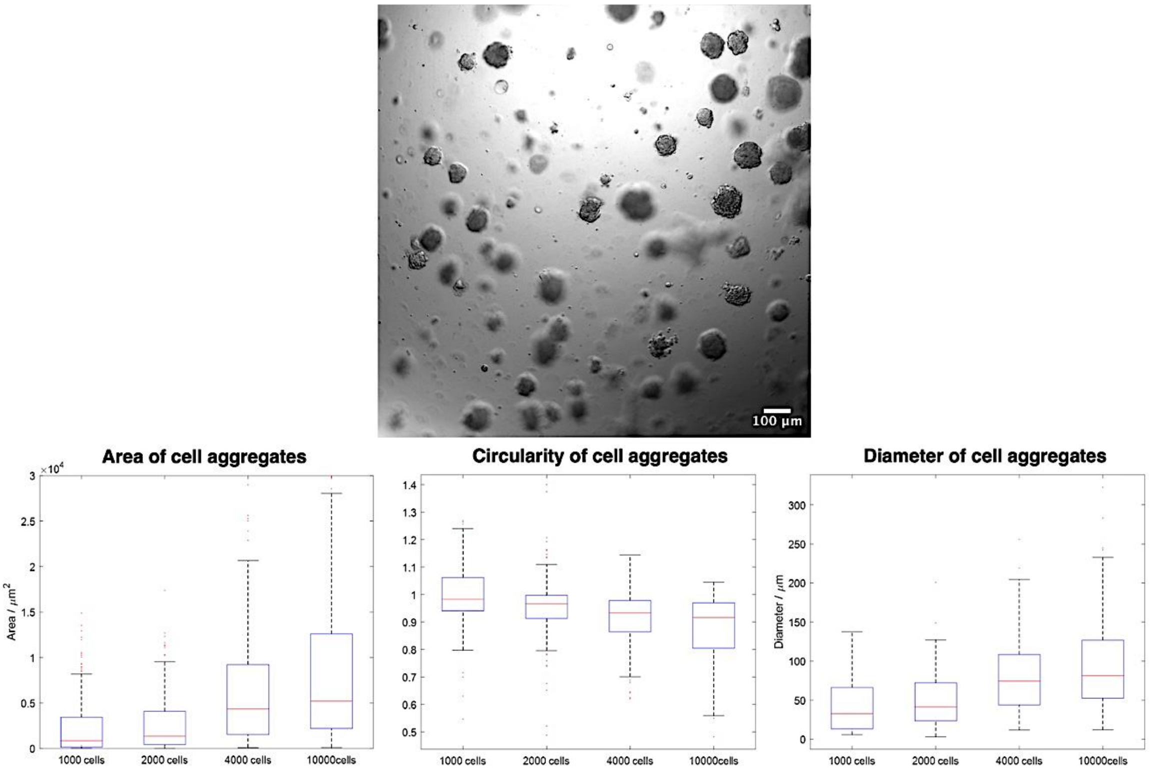


**Fig.S3: HCT116 spheroids generated with scaffold-based method.** (Top) Representative image of MTS cultured for 7 days in Basement Membrane Matrix (1000 cells/well, scale bar 100 µm). (Bottom) Area, circularity and diameter analysis of spheroids obtained by seeding 1000, 2000, 4000, 10000 cells/well) n=200.

## S4: Comparison of growth rates in agarose and PDMS culture microwells devices

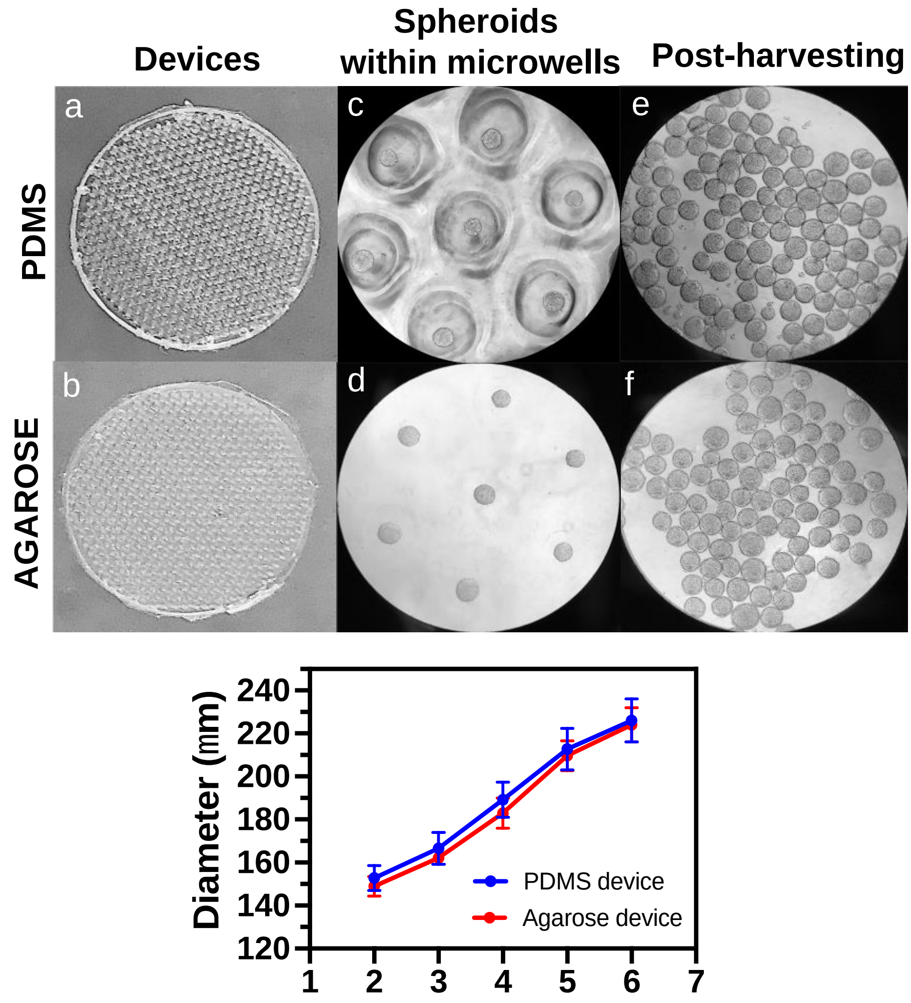


**Fig.S4: Agarose devices as alternative material for spheroids production** (a, b) Low magnification images of PDMS and agarose devices surface. (c,d) Spheroids array in microwells imaged with 10X lens. (e,f) The harvested cell spheres were intact and uniform with both device materials. The graph on the bottom shows growth curves comparison, data relative of 28 spheroids per different device (n=3).

**S5: Comparison of the prototype elastomeric devices with commercial microwell culture devices.**

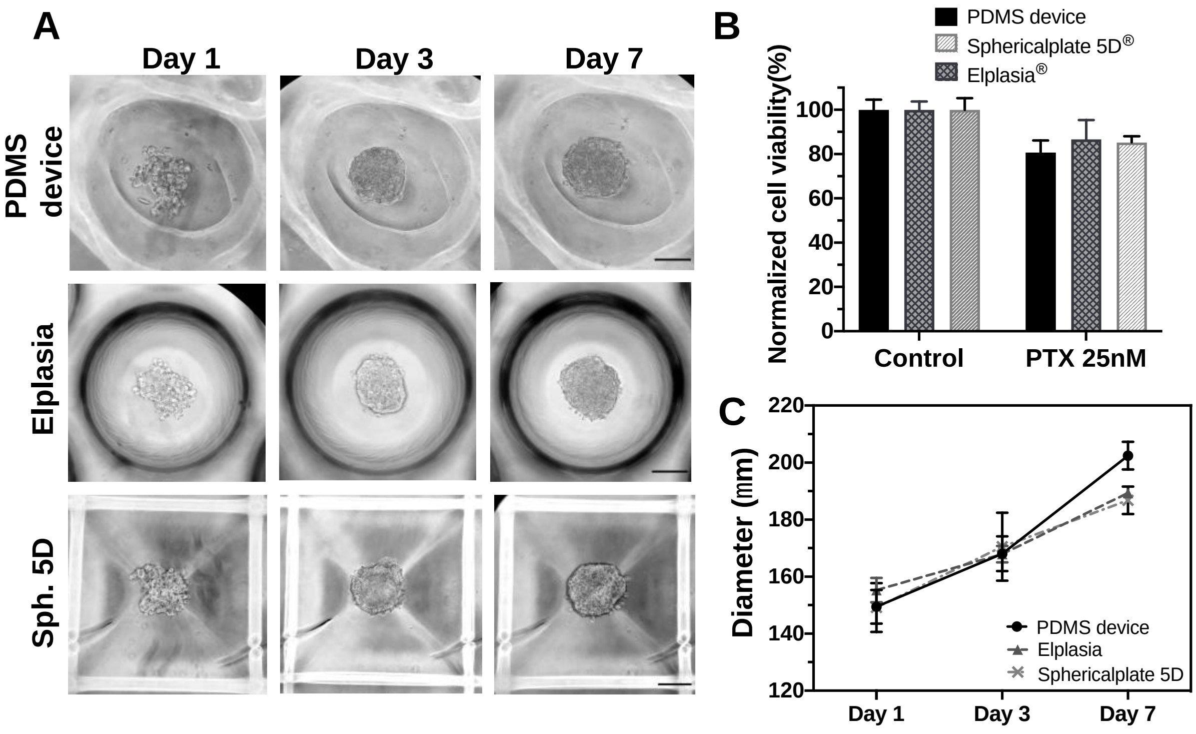


**Fig.S5: Comparison of HCT116 spheroids produced with prototype device and commercial culture plates.** (A) Phase contrast images of HCT116 spheroids initiated with 150 cells/microwell on the three platforms, scale bar: 100 μm. (B) Effect of Paclitaxel treatment on spheroids viability. Colon cancer spheroids demonstrated reduced viability in response to Paclitaxel treatment. Viability of treated spheroids did not change significantly depending on the culture platform (one-way ANOVA). Viability within spheroids was measured using MTT assay and normalized to untreated control spheroids generated on the same platform. (C) Diameter measurement of HCT116 spheroids generated on prototype device, Elplasia and Sphericalplate 5D. Curves represent quadruplicate biological repeats and are displayed as mean ± SEM (n = 4).

**S6: MTS growth in scaffolded or non-scaffolded conditions for MCF-7 cells.**

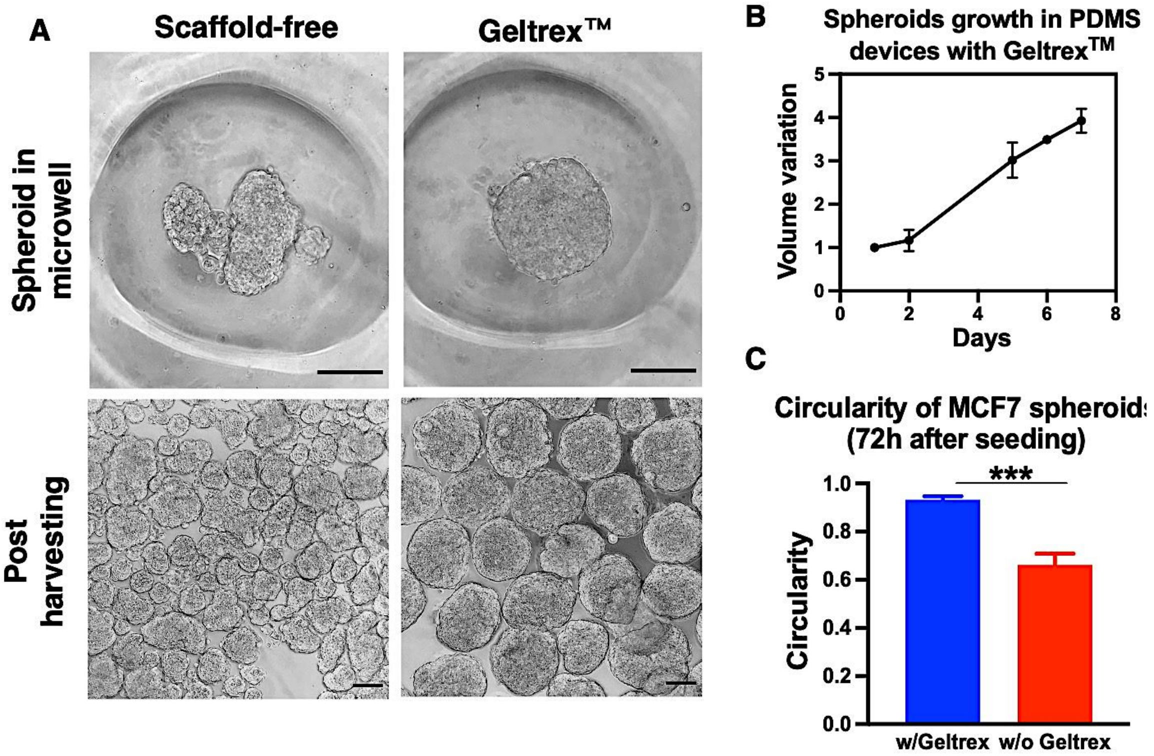


**Fig.S6: MCF-7 spheroids assembly in prototype inserts with Geltrex$^{TM}$.**
(A) Representative brightfield images of MCF-7 spheroids cultured in PDMS device with and without use of Basement Membrane Matrix, within microwells and after spheroids collection (scale bar: 100µm). (B) MCF-7 spheroids growth curve. (C) Mean circularity of MCF-7 spheroids cultured in three different devices changed significantly depending on the method applied for spheroid generation (difference between means of 0.27 ± 0.01; n=20).

**S7: The response of MTS to the treatment with cytotoxic drugs: dependence on the size of MTS at the onset of treatment, as controlled with the seeding density**
.

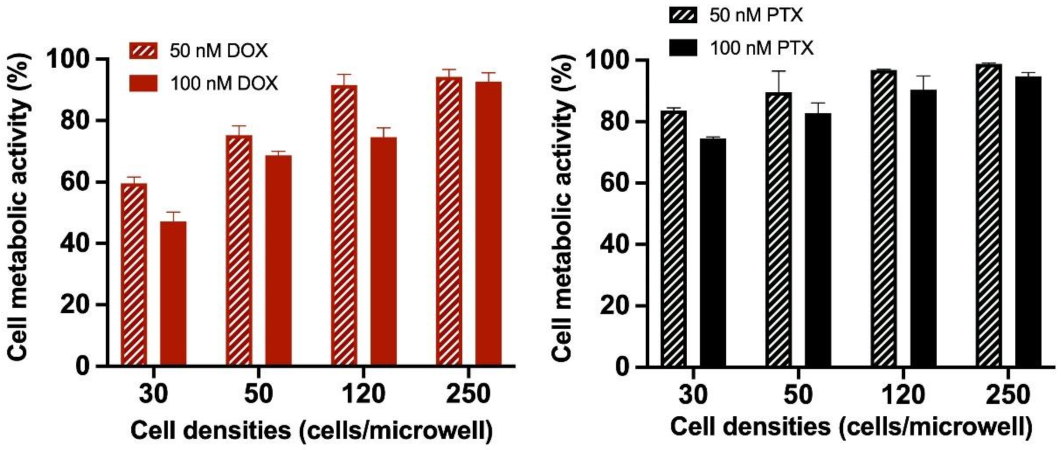


**Fig.S7: Cell viability histograms of different sized tumor spheroids after DOX and PTX treatment.** HCT116 tumor spheroids in different sizes were formed on the prototype device after seeding 30, 50, 120, 250 cells/microwell. Then the spheroids were treated with DOX (left) and PTX (right) for 24 h and the cell viability was analyzed using MTT assay. The error bars represent the standard error from three independent experiments.

**S8: Experimental comparison of the response of 2D and 3D cell cultures (MTS) to the treatment with cytotoxic drugs.**

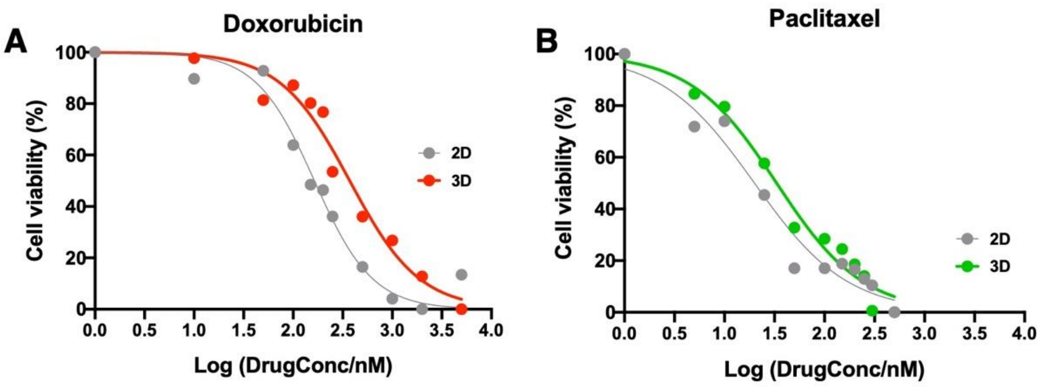


| $IC_{50}$ values | Doxorubicin | Paclitaxel |
|---|---|---|
| 2D | 132 nM (± *24*) | 30 nM (± *6*) |
| 3D | 280 nM(± *80)* | 38 nM (± *3*) |
| 3D/2D | 215% | 122% |

**Figure S8: 3D vs. 2D comparison for the effect of treatment with cytotoxic drugs and the determination of $IC_{50}$.** Drug effects of Doxorubicin (A) Paclitaxel (B) in spheroids cultures, with comparison to monolayer cell cultures. Dose-response curves were plotted exponentially

**S9: Characterization of the physical properties of spheroids cultured in prototype device (W8 analysis, CellDynamics)**

W8 (CellDynamics SRL) is a flow-apparatus balance that can measure the size, weight, and density of individual spheroids simultaneously and accurately (physical cytometer). The measurement is based on the terminal velocity of a free-falling microscale object (such as a spheroid) in the instrument's flow-channel. The physical approach to this analysis, as well as the mathematical equations used for calculation, are fully described by Cristaldi et al. 2020 [1]. Courtesy of CellDynamics, we analyzed two specimens: control SKOV-3 spheroids and SKOV-3 spheroids treated with PTX 25nM. Spheroids were cultured and harvested from our PDMS devices.
It was observed that the PTX treatment influenced spheroid mass density: a statistical difference was found between control (1031.1 ± 2.2 fg/μm$^3$) and treated spheroids (1022.2 ± 3.0 fg/μm$^3$) of the SKOV-3 cell line. The diameter evaluation of the spheroid culture generated in PDMS devices confirmed the morphological data discussed in this paper. In fact, control spheroids were characterized by a diameter of 227 ± 11 μm and a narrow size distribution range. Additionally, as expected, it was observed that the diameter of PTX treated spheroids was reduced of about 50 μm and that the sample presented a broader values range of 174 ± 27 μm. A statistical difference was found between the weights of the two spheroids populations. Control spheroids resulted to weight 6.3 ± 0.9 μg, while the weight of treated spheroids decreased to 3.0 ± 1.2 μg.
Our produced (control) spheroids turned out to be similarly or more homogeneous than other specimens characterized by the same instrument and reported in the literature. For example, Cianciosi et al. showed spheroids with a 14% dispersion in their diameter (SD/average) and a weight dispersion of 40%, while for ours these values were 5% and 14%, respectively [2].

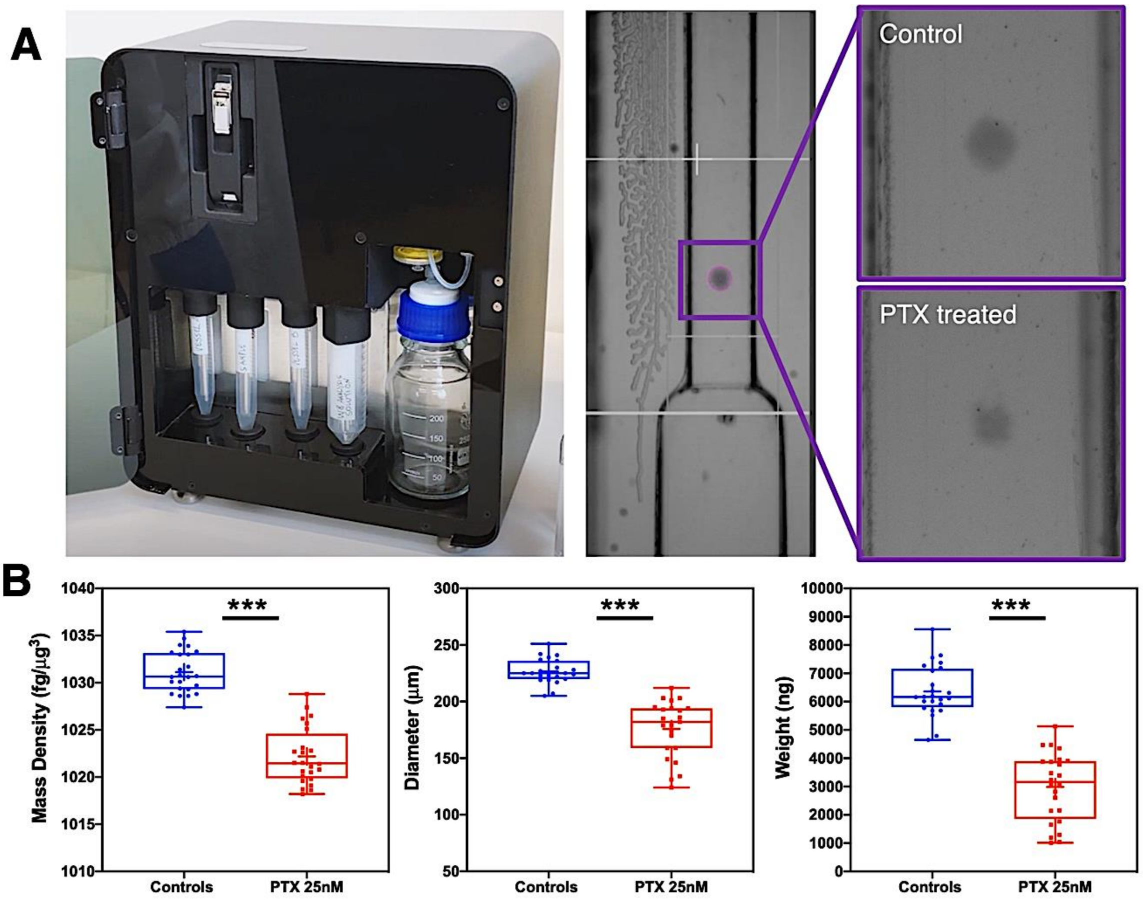


**Figure S9: W8 automated platform and fluidic core-chip for the characterization of the physical properties of spheroids** (A) Photographs of the W8 physical cytometer. On the right are example images of frames collected for two representative samples. A large and rounded control spheroid in the top right panel, and a medium sized treated spheroid in the bottom right panel, showing a slight deviation from sphericity. (B) Box-and-whisker plots exhibiting the distribution of mass density, diameter, and weight for every condition under testing. The lines, extending from the boxes, indicate variability outside the upper and lower quartiles. Results are expressed as the mass density (fg/µm$^3$, left graph), diameter (µm, central graph) and weight (ng, right graph). The mean value and SD of these three parameters (MD, W, D) are calculated for each sample. Student's t-test (two-tailed and heteroscedastic) was used to assess the statistical significance between the two data sets: p-value < 0.001 (***) , n=24 per group.

**S10: wide field imaging of live-dead stained, untreated or treated MTS while still arranged as in the culture microwell.**

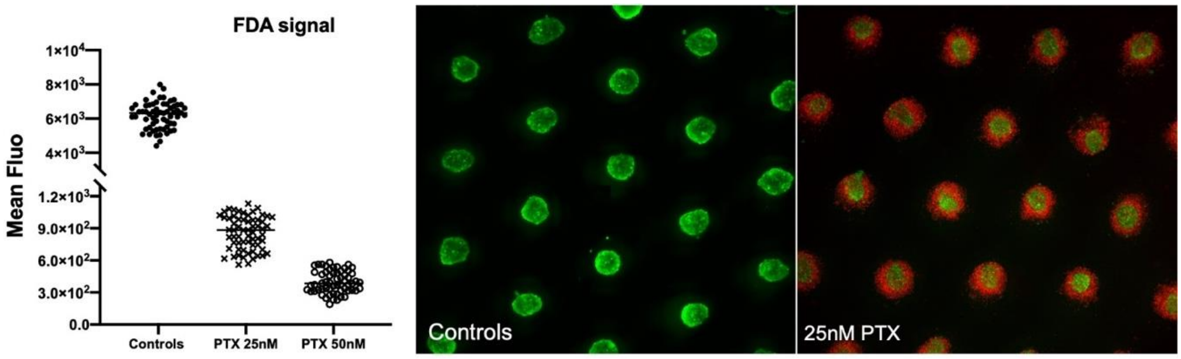


**Figure S10: Spheroids treated in PDMS device showed reproducible fluorescence signals.** On the left, individual fluorescein diacetate (FDA) fluorescence signals of HCT116 spheroids control and treated with 25nM and 50nM PTX. Coefficient variation% of 12%,18% and 26% respectively (n=60 per group). Middle and right images showing MTS incubated with fluorescein diacetate(green) and propidium iodide (red), 5X magnification.

**S11: Confocal microscopy imaging of immunofluorescence staining of MTS as originally arranged in the culture microwells array.**

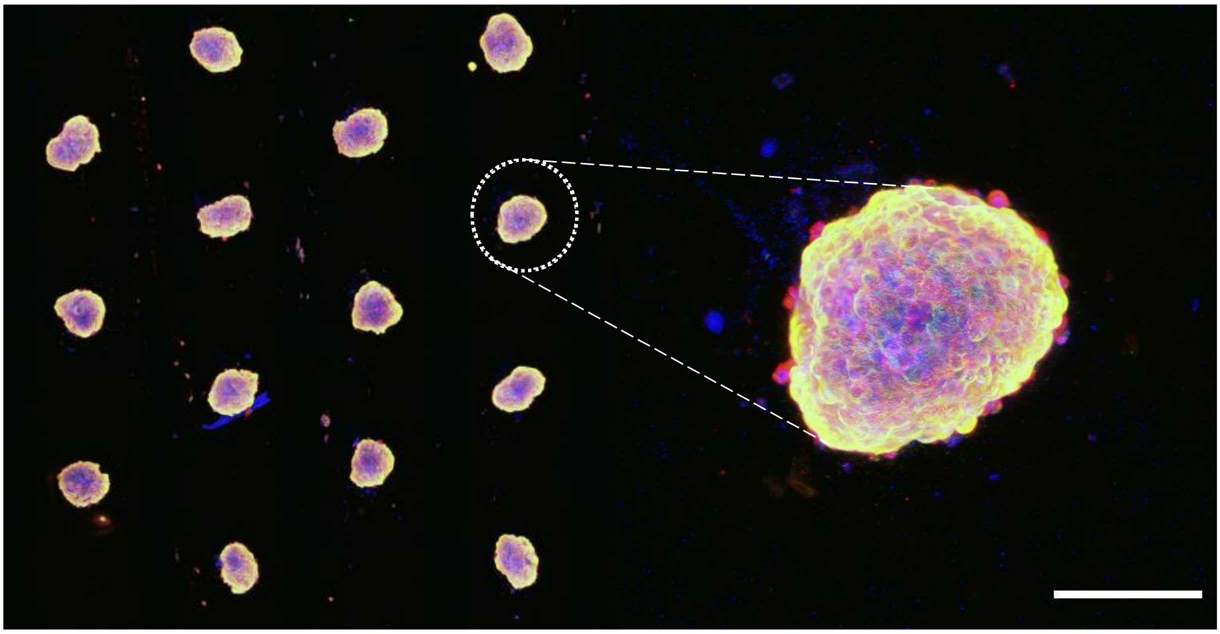

**Figure S11:** Maximal Image Projection (MIP) of confocal fluorescence images of spheroids in prototype device labelled for beta-actin (green), alpha-tubulin (red) and nuclei (blue) (20X magnification) and an enlarged MIP of one of the spheroids (40X magnification, scale bar 100 µm).

## SI_Video_1.mp4

**Supplementary Video 1 description:** 24 hours time-lapse video of HCT116 spheroids formation (~50 microwells/field). Immediately after seeding (50 cells/microwell), single cells within microwells cluster and spheroid formation begins. Automatic device monitoring was performed with CytoSMART$^{TM}$ Omni.

### Supplementary Information Bibliography


1. Cristaldi DA, Sargenti A, Bonetti S, et al (2020) A Reliable Flow-Based Method for the Accurate Measure of Mass Density, Size and Weight of Live 3D Tumor Spheroids. Micromachines 11:465

2. Cianciosi D, Forbes-Hernández TY, Regolo L, Alvarez-Suarez JM, Quinzi D, Sargenti A, Bai W, Tian L, Giampieri F, Battino M (2022) Manuka honey in combination with 5-Fluorouracil decreases physical parameters of colonspheres enriched with cancer stem-like cells and reduces their resistance to apoptosis. Food Chem 374:131753
